# Genome-wide significant risk factors on chromosome 19 and the *APOE* locus

**DOI:** 10.18632/oncotarget.25083

**Published:** 2018-05-15

**Authors:** Sonia Moreno-Grau, Isabel Hernández, Stefanie Heilmann-Heimbach, Susana Ruiz, Maitée Rosende-Roca, Ana Mauleón, Liliana Vargas, Octavio Rodríguez-Gómez, Montserrat Alegret, Ana Espinosa, Gemma Ortega, Nuria Aguilera, Carla Abdelnour, Alzheimer’s Disease Neuroimaging Initiative, Silvia Gil, Wolfgang Maier, Oscar Sotolongo-Grau, Lluís Tárraga, Alfredo Ramirez, Jesús López-Arrrieta, Carmen Antúnez, Manuel Serrano-Ríos, Mercè Boada, Agustín Ruiz

**Affiliations:** ^1^ Research Center and Memory Clinic of Fundació ACE, Institut Català de Neurociències Aplicades, Univesitat Internacional de Catalunya, Barcelona, Spain; ^2^ Institute of Human Genetics, University of Bonn, Bonn, Germany; ^3^ Department of Genomics, Life & Brain Center, University of Bonn, Bonn, Germany; ^4^ Department of Psychiatry and Psychotherapy, University of Bonn, Bonn, Germany; ^5^ German Center for Neurodegenerative Diseases, DZNE, Bonn, Germany; ^6^ Department of Psychiatry and Psychotherapy, University of Cologne, Cologne, Germany; ^7^ Memory Unit, University Hospital La Paz-Cantoblanco, Madrid, Spain; ^8^ Dementia Unit, University Hospital Virgen de la Arrixaca, Murcia, Spain; ^9^ Centro de Investigación Biomédica en Red de Diabetes y Enfermedades Metabólicas Asociadas, CIBERDEM, Spain, Hospital Clínico San Carlos, Madrid, Spain

**Keywords:** late onset Alzheimer’s disease, ABCA7, APOE, CD33, linkage disequilibrium, Gerotarget

## Abstract

The apolipoprotein E (*APOE*) gene on chromosome 19q13.32, was the first, and remains the strongest, genetic risk factor for Alzheimer’s disease (AD). Additional signals associated with AD have been located in chromosome 19, including *ABCA7* (19p13.3) and *CD33 (*19q13.41). The *ABCA7* gene has been replicated in most populations. However, the contribution to AD of other signals close to *APOE* gene remains controversial. Possible explanations for inconsistency between reports include long range linkage disequilibrium (LRLD). We analysed the contribution of *ABCA7* and *CD33* loci to AD risk and explore LRLD patterns across *APOE* region. To evaluate AD risk conferred by *ABCA7* rs4147929:G>A and *CD33* rs3865444:C>A, we used a large Spanish population (1796 AD cases, 2642 controls). The *ABCA7* rs4147929:G>A SNP effect was nominally replicated in the Spanish cohort and reached genome-wide significance after meta-analysis (odds ratio (OR)=1.15, 95% confidence interval (95% CI)=1.12–1.19; *P* = 1.60 x 10^-19^). *CD33* rs3865444:C>A was not associated with AD in the dataset. The meta-analysis was also negative (OR=0.98, 95% CI=0.93–1.04; *P*=0.48). After exploring LRLD patterns between *APOE* and *CD33* in several datasets, we found significant LD (D’ >0.20; *P* <0.030) between *APOE*-Ɛ2 and *CD33* rs3865444C>A in two of five datasets, suggesting the presence of a non-universal long range interaction between these loci affecting to some populations. In conclusion, we provide here evidence of genetic association of the *ABCA7* locus in the Spanish population and also propose a plausible explanation for the controversy on the contribution of *CD33* to AD susceptibility.

## INTRODUCTION

The development of novel approaches in genetics has led to the identification of 30 genetic determinants of Late Onset Alzheimer’s disease (LOAD) [1]. The most important risk locus for LOAD remains on the long arm of chromosome 19 containing the *APOE* locus, which was the first, and is the strongest, risk factor for Alzheimer’s disease (AD) [2]. Three *APOE* diplotypic alleles (Ɛ2, Ɛ3, and Ɛ4) are defined, which result from the combination of two single nucleotide polymorphisms (SNPs), rs7412:C>T and rs429358:C>T. While the Ɛ2 allele of *APOE* is determined by the minor allele of rs7412:C>T and is protective for LOAD [3], the minor allele of rs429358:C>T defines the Ɛ4 allele, which increases the risk of LOAD [2] by up to four-fold.

Interestingly, additional loci on chromosome 19 have been proposed, namely *TOMM40* [4], *EXOC3L2* [5], *CD33* [6], *PLD3* [7], and *ABCA7* [6] [8]. While compelling genetic data have confirmed *ABCA7*, on the short arm of chromosome 19, as an *APOE*-independent AD risk factor [9, 10], inconsistent results have been reported for the other four genes, after adjustments for the effects of *APOE* [11, 12]. Particular interest deserves the *CD33* locus which was reported in 2011 using meta-analysis of genome-wide association studies (GWAS) [6] and, its effect was nominally replicated in an additional sample [13]. However, the association signal of *CD33* did not reach genome-wide significance in the International Genomics Alzheimer’s Project (IGAP) meta-analysis [9], the largest case-control study for LOAD. Thus, further investigations are required to confirm or refute the potential association of *CD33* with LOAD.

Several possibilities may explain the inconsistency in the results of genetic association studies of regions around the *APOE* locus such as *CD33*, including the large contribution of the *APOE* allele to AD risk, the existence of long range linkage disequilibrium (LRLD) regions in different populations, the existence of hidden familial cases and population inbreeding. However, a definitive explanation for the real driving-force behind these findings is lacking.

Thus, we explored the genetic contribution of *ABCA7* and *CD33*, including their interactions with *APOE*, to AD risk in a large independent AD case-control cohort composed by unrelated individuals who have at least two Spanish ancestors reported. Furthermore, we conducted LRDL analyses across several dataset to evaluate its influence on *CD33* association to AD.

## RESULTS

### Replication results and meta-analysis

**Figure 1:**
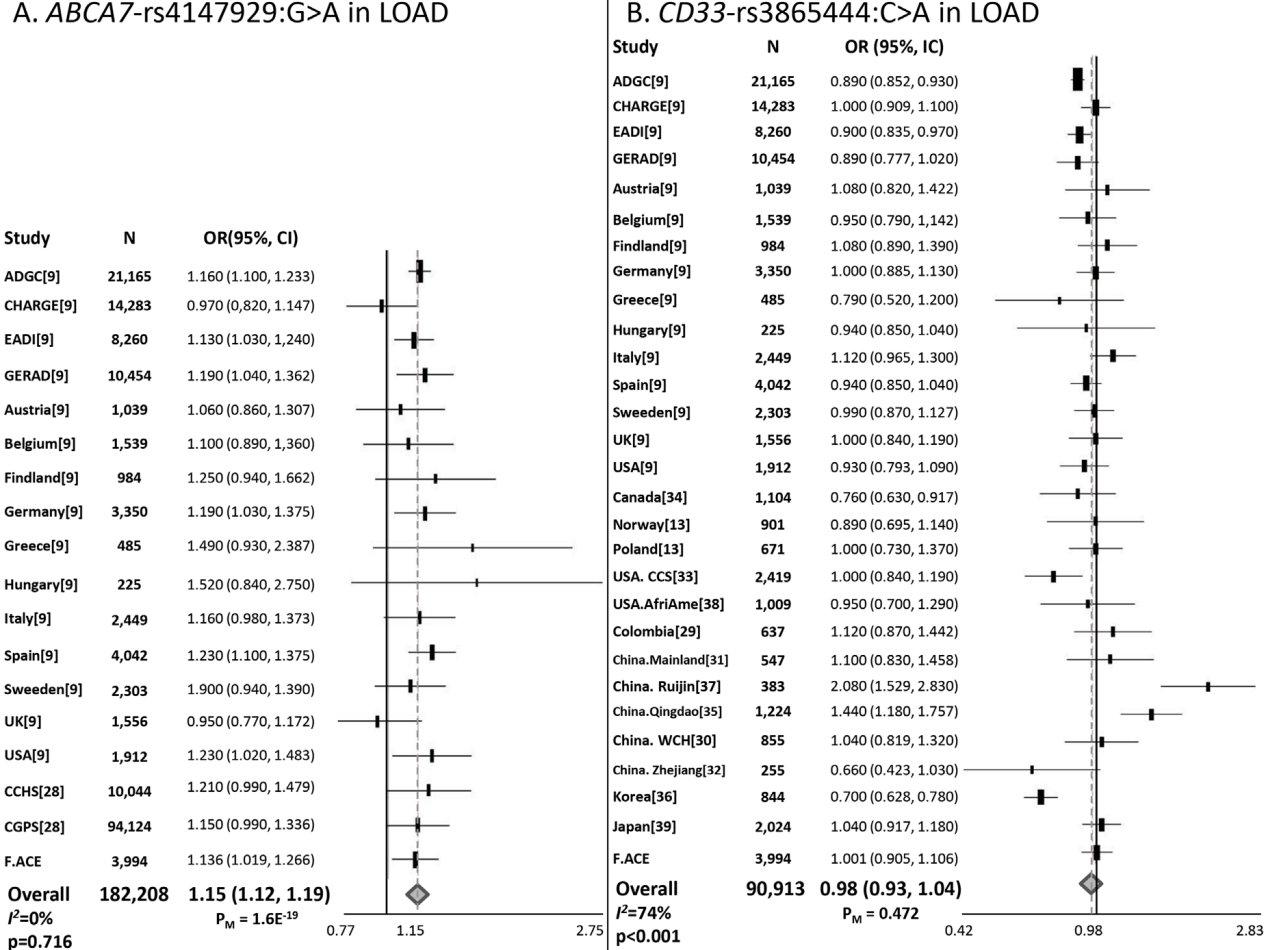
Forest plots for **(A)** *ABCA7* rs4147929:G>A and **(B)** *CD33* rs3865444:C>A. *ABCA7* rs4147929:G>A and *CD33* rs3865444:C>A meta-analyses comprised data from the IGAP datasets, independent replications, and the present study.

SNPs included in this study were in Hardy-Weinberg equilibrium (*P* < 0.05). A significant risk effect was observed for the minor allele of *ABCA7* rs4147929:G>A (OR=1.15; 95% CI=1.04–1.27) in the Spanish population. *ABCA7* effect survived co-variation and *APOE*-Ɛ4 stratification analyses (Table 1). Meta-analysis including new data generated, the IGAP results and studies fulfilling inclusion criteria (n = 182 208), re-affirmed a genome-wide significant association for *ABCA7* (OR=1.15; 95% CI=1.12–1.19, Figure 1A). For *CD33*, we could not replicate the originally reported protective effect of rs3865444:C>A SNP. In the *APOE*-Ɛ4 stratified analysis, *CD33* did not modulate susceptibility to AD in any stratum (Table 1). Our meta-analysis also revealed heterogeneity and a non-significant association (Figure 1B).

**Table 1:** Association between *ABCA7* rs4147929:G>A or *CD33* rs3865444:C>A and LOAD in unadjusted, adjusted and *APOE*-Ɛ4 stratified models

GENE marker	Unadjusted model	Adjusted (sex and APOE- ε4)	Adjusted (age, sex and APOE-ε4)	Stratification per APOE-ε4	
				Carriers	Non- Carriers
**ABCA7** **rs4147929**	OR =1.148 CI =1.036 – 1.27 p = 0.008	OR = 1.190 CI = 1.070 – 1.324 p = 0.0013	OR = 1.175 CI = 0.94 – 1.47 p = 0.1546	OR = 1.225 CI = 1.006 – 1.492 p = 0.043	OR = 1.167 CI = 1.028 – 1.324 p = 0.017
**CD33** **rs3865444**	OR = 0.986 CI = 0.897 – 1.084 p = 0.771	OR = 0.976 CI = 0.885-1.076 p = 0.6287	OR = 0.920 CI = 0.750 –1.127 p = 0.4182	OR = 1.157 CI = 0.970 – 1.380 p = 0.104	OR = 0.899 CI = 0.797 – 1.014 p = 0.083

Abbreviations: LOAD, Late Onset Alzheimer’s disease; OR, Odds Ratio; CI, Confidence Interval.

### Linkage disequilibrium analysis

Next, we sought to explore potential reasons for the lack of replication of the association of *CD33* rs3865444:C>A with AD in some populations. Specifically, patterns of LD were studied in five datasets, comprising data from the present study and four non-imputed GWAS datasets.

We detected significant LRLD between *APOE* rs7412:C>T and *CD33* rs3865444:C>A in two of the five datasets analysed (D’ ≥ 0.2) (Table 2). The probability of these results being spurious was 0.011 for the Murcia dataset and 0.002 for the NIA data (Supplementary Table 1). However, the other datasets did not display LRLD: ADNI, D’ = 0.17 (*P* = 0.11); GenADA, D’ = 0.14 (*P* = 0.004); and F.ACE, D’ = 0.01 (*P* = 0.86) (Table 2). In contrast, only one dataset exhibited significant LD between *APOE* rs429358:C>T and *CD33* rs3865444:C>A; however it did not reach the lower threshold for LD (D’ ≥ 0.2).

**Table 2:** Linkage disequilibrium results and test of significance for LD between *APOE* rs7412:C>T or rs429358:C>T and *CD33* rs3865444:C>A

Dataset	LD				
	N	D’	r2	Chi sq	P value
***APOE* rs429358:C>T – *CD33* rs3865444:C>A**					
**ADNI**	358	−0.087	0.001	0.86	0.350
**GenADA**	1555	−0.105	0.001	5.32	**0.020**
**Murcia**	1088	+ 0.036	5.00e-04	1.16	0.280
**NIA**	1789	+ 0.015	2.00e-04	0.75	0.380
**F_ACE**	4402	+ 0.013	8.00e-05	0.70	0.400
***APOE* rs7412:C>T – *CD33* rs3865444:C>A**					
**ADNI**	358	+ 0.174	0.003	2.62	0.105
**GenADA**	1555	+ 0.138	0.002	8.00	**0.004**
**Murcia**	1088	−0.312	0.002	5.75	**0.016**
**NIA**	1789	−0.228	0.001	4.70	**0.030**
**F_ACE**	4402	+ 0.005	3.25e-06	0.03	0.860
***APOE* rs429358:C>T – *APOE* rs7412:C>T**					
**ADNI**	358	−1	0.020	13.79	**2.00e-04**
**GenADA**	1555	−1	0.020	63.78	**1.44e-15**
**Murcia**	1088	−1	0.010	23.42	**1.30e-06**
**NIA**	1789	−1	0.030	93.22	**4.68e-22**
**F_ACE**	4402	−1	0.010	91.06	**1.39e-21**

**Figure 2:**
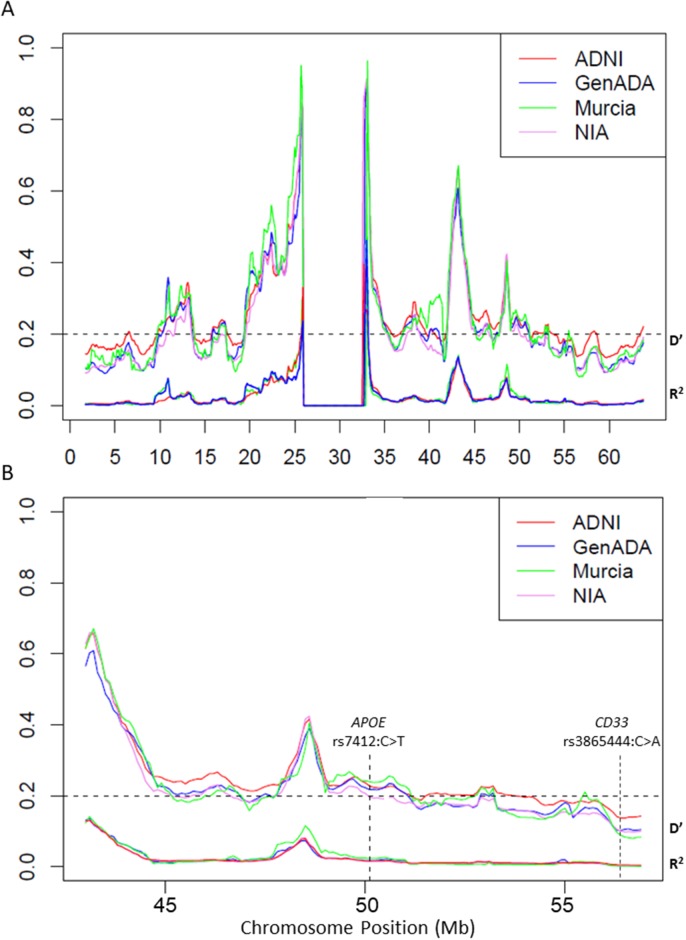
Linkage disequilibrium patterns across **(A)** chromosome 19 and **(B)** *APOE* region. Average D’ (top groups) and r^2^ coefficients (bottom groups) plotted in sliding windows containing all common polymorphisms separated by 50 and 500 kb in successive 1.7-Mb segments (1.6-Mb overlap). Genome assembly NCBI36/hg18.

We next tested the LRLD hypothesis by assessing the pattern of LD along chromosome 19. This alternative strategy highlighted common areas of high LD in all datasets, particularly at positions 10.7–13.5 Mb, 42–45.1 Mb, and 47.8–50 Mb (Figure 2A). In addition, the Murcia and ADNI dataset exhibited the largest segments of LD at positions 9.7–10.7Mb and 9.8–13.8 Mb, respectively. ADNI dataset also presents the largest LD segment at positions 41.8–51.1Mb, the latter of which contained the *APOE* gene at position 50.1 Mb (Figure 2B). However, this strategy did not reveal a general LD between *APOE* and *CD33.* This observation is supporting the notion that long range LD between *CD33* and *APOE*, if exist, would be affecting to a discrete fraction of chromosomes in some human populations.

Finally, an association analysis approach according to the presence or absence of the *APOE* Ɛ2 genotype also supported that long range association between *APOE* and *CD33* is not very common in studied populations. After Bonferroni correction, the majority of significant signals mapped to a region of chromosome 19, 231 kb upstream of *APOE* rs7412C>T (Supplementary Table 2).

### *APOE* stratification analysis and meta-analysis of GWAS data

Stratification analysis revealed a significant protective association for *CD33* rs3865444:C>A only in Ɛ4 allele carriers in the GenADA dataset (OR = 0.69; *P* = 0.009) (Table 3). This protective trend for *CD33* rs3865444:C>A was also observed in Ɛ2 carriers of Spanish origin (Murcia study: OR = 0.49, 95% CI, 0.19–1.25; F.ACE: OR = 0.68, 95% CI 0.45–1.03). Interestingly, the minor allele of *CD33* rs3865444:C>A was associated with increased susceptibility to AD in Ɛ4 carriers in the same Spanish datasets (Murcia study: OR = 1.13, 95% CI, 0.79–1.63; F.ACE: OR = 1.15, 95% CI, 0.97–1.37). Opposite effect directions were observed in the NIA and ADNI datasets. After meta-analysis, homozygous Ɛ3Ɛ3 carriers were identified as the most homogeneous stratum for *CD33* effects, while Ɛ4 allele carriers were the most heterogeneous (Table 3). Finally, we excluded hidden relatedness and population stratification as a potential cause for LRLD by analysing inbreeding (Supplementary Table 3) and conducting principal components (PCs) analysis across the datasets (Supplementary Figure 1).

**Table 3:** Stratification per Ɛ2, Ɛ4 *APOE* alleles and Ɛ3Ɛ3 *APOE* genotype for five studied dataset and meta- analysis results per stratums

	Unadjusted model	Ɛ2 allele carriers	Ɛ4 allele carriers	Ɛ3Ɛ3 genotype carriers
**ADNI**	OR = 0.865 CI95% = 0.627 –1.193 p = 0.377	OR = 2.2 CI95% = 0.553 – 8.76 p = 0.256	OR = 0.979 CI95% = 0.580 – 1.654 p = 0.938	OR = 0.706 CI95% = 0.419 – 1.187 p = 0.188
**GenADA**	OR = 0.864 CI95% = 0.742 – 1.006 p = 0.06	OR = 0.863 CI95% = 0.483 – 1.544 p = 0.620	OR = 0.697 CI95% = 0.540 – 0.899 p =**0.005**	OR = 1.085 CI95% = 0.862 – 1.366 p = 0.487
**Murcia**	OR = 0.938 CI95% = 0.763 – 1.155 P = 0.550	OR = 0.492 CI95% = 0.193 – 1.252 p = 0.130	OR = 1.133 CI95% = 0.788 – 1.629 p = 0.498	OR = 0.849 CI95% = 0.635 – 1.135 p = 0.269
**NIA**	OR = 0.956 CI95% = 0.829 – 1.103 p = 0.540	OR = 1.356 CI95% = 0.660 – 2.784 p = 0.401	OR = 0.873 CI95% = 0.691 – 1.104 p = 0.257	OR = 0.908 CI95% = 0.714 – 1.154 p = 0.430
**F.ACE**	OR = 0.986 CI95% = 0.897 – 1.084 p = 0.772	OR = 0.682 CI95% = 0.453 – 1.027 p = 0.066	OR = 1.153 CI95% = 0.967 – 1.376 p = 0.112	OR = 0.928 CI95% = 0.818 – 1.054 p = 0.25
**Meta-Analysis**	OR = 0.947 CI95% = 0.887 – 1.010 p = 0.098	OR = 0.850 CI95% = 0.650 – 1.111 p = 0.234	OR = 0.946 CI95% = 0.765 – 1.170 P = 0.609	OR = 0.932 CI95% = 0.849 – 1.023 p = 0.141
**Heterogeneity**	*I*^2^ = 0% p = 0.658 Fixed	*I*^2^ = 32% p = 0.206 Fixed	*I*^2^ **= 66%** p = **0.020** Random	*I*^2^ = 0% p = 0.523 Fixed

## DISCUSSION

Confirmatory data on AD associations with loci neighbouring *APOE* are still lacking, despite several replication efforts. In our work, we clearly replicated the *ABCA7* signal (*P* = 1.60 x 10^-19^), which is located far from the *APOE* locus. However, we failed to replicate the *CD33* association in the Spanish population. Both observations are independent and consistent with previous IGAP observations [9].

*ABCA7* marker was, firstly, identified such an AD locus by Hollingworth et. al. [6] and Naj et al. [8], in a large cohort of individuals (n=29,544). Following, this association was supported by IGAP consortium [9] and confirmed in non-European populations [10]. Our finding is in accordance with previously studies. It reinforces the role of *ABCA7* as a candidate gene for AD and warrants its functional characterisation. *ABCA7* is involved in lipid metabolism [14] and apoptotic cell clearance [15]. Its loss has been related to deficient macrophage clearance of amyloid plaques [16] and to accelerated amyloid-beta production [14].

*CD33* locus appeared such as a promising signal [6] especially as a candidate target for immune-related therapies [17]. Despite that, the signal disappeared after the IGAP meta-analysis [9], casting doubts on its real contribution to AD. In that scenario, we believed that the *CD33* locus appears to be affected by the “*APOE* curse”; i.e., the impossibility of determining whether additional AD loci truly exist around *APOE*. Similar to *CD33*, conflicting data exist for *TOMM40*’523, *EXOC3L2*-597668, and *PLD3* (V232M) [11, 12]. Several strategies have been attempted to tackle this issue; for example, using phylogenetic analysis, the *TOMM40*’523 poly-T marker was shown to be associated with age-at-onset in the *APOE* Ɛ3 subgroup [4]. However, this finding has not been extensively replicated [18].

The lack of association between AD and *CD33* prompted us to search for an underlying explanation. Our data suggested significant, weak and non-universal, LRLD between the *APOE-*Ɛ2 allele and *CD33* rs3865444:C>A. Of course, there are limitations on these observations. First of all, very low levels of *r2* were detected, however the intrinsic properties of *r2* prompts to consider *D’* such as more informative measure for assessing historical recombination in a given population [19]. Furthermore, we are dealing with relatively weak *D’* values, roughly 0.3, which means that 30% of the chromosomes will be carrying the long LD tract in specific populations. This could be compromising the capacity to detect LRLD when a direct measure of LD between studied markers is not determined.

Second, LRLD patterns differ across populations and are dependent on many factors, including admixture or migration, genetic drift, chromosome inversions, epistatic selection and hitchhiking effects [20]. Furthermore, differential natural selection pressures across genomic regions, depending on specific geographical or environmental conditions, can lead to differential patterns of allele micro-heterogeneity. Although differential population structure was not identified in our GWAS datasets analysis, there is compelling evidence that micro-stratification cannot be detected by standard methods [21] and, therefore, this remains a potential limitation. Thus, the existence of undetected population sub-structure, with different LRLD patterns, could act as confounding factor, explaining the divergent observations and lack of replication between *CD33* and AD across studies [9]. Of note, the genuine or spurious character of the association would remain masked under LRLD patterns, being its appearance highly dependent of the population structure.

An additional explanation for the lack of association between *CD33* and AD could be that the original *CD33* signals were simply chance findings. We feel that this possibility is less likely because under the assumption of a random association between *CD33* and AD, the chance of observing effects in opposite directions in independent studies would have the same probability. This latter observation is clearly not the situation reported in the literature to date.

Importantly, we detected LRLD upstream of the *APOE* locus using a previously described method [22]. Confirmation of LRLD around *APOE* is relevant for several reasons. First, the data will assist in clarifying whether or not reported AD signals are genuine. Second, differential LRLD across populations may reveal the existence of structural variations, such as large inversions, insertions, or deletions, unequally affecting human populations. Structural variants have been implicated in the aetiology of the majority of multifactorial diseases [23].

In summary, we confirm *ABCA7* is associated with LOAD. However, we could not confirm the association between *CD33* and AD. Our data suggest that LRLD between *CD33* markers and the *APOE* alleles might explain the observed lack of consistency of *CD33* signal. Further studies using independent populations are required to clarify whether LRLD interferes with real associations between loci around *APOE* and AD.

## MATERIALS AND METHODS

### Replication study in the spanish population: subjects and genotyping

The Spanish sample comprised 1796 unrelated sporadic AD patients (mean age, 82.1 ± 7.9 years, 70.2% women) and 2642 healthy controls (mean age, 54.1 ± 11.6 years, 64.3% women) recruited at Fundació ACE, Institut Català de Neurociències Aplicades (Barcelona, Spain); Unidad de Memoria, Hospital Universitario La Paz-Cantoblanco (Madrid, Spain); Hospital Clínico Sán Carlos Unidad de Demencias, Hospital Universitario Virgen de la Arrixaca (Murcia, Spain) and Neocodex S.L. (Supplementary Table 4). Sample characteristics were previously described by Antunez et al. [24]. Briefly, all AD patients fulfilled Diagnostic and Statistical Manual of Mental Disorders IV criteria for dementia and were diagnosed according to National Institute of Neurological and Communicative Disorders and Stroke and the Alzheimer’s disease and Related Disorders Association criteria for possible and probable AD. Ethics committees from each referral centre approved the research protocol. All participants provided written informed consent.

Standard methods were used to isolate DNA. The SNP rs4147929:G>A, this is hg19 chr19:g.1063444A>G located in the *ABCA7* gene and the SNP rs3865444:C>A, this is hg19 chr19:g.51727962C>A located in *CD33* gene, were genotyped using Sequenom technology (Sequenom, California, USA), as previously described [25]. Primer sequences and assay conditions for the genotyped SNPs are available upon request.

*APOE* rs7412C>T and rs429358:C>T markers were genotyped using real-time PCR. Primers design was previously described by Calero et al [26]. Briefly, PCR reactions were performed in a final volume of 5μl, using 11 ng of genomic DNA, 0.3 μM of each amplification primer and 2.65μl of 2X SYBR Fast Master Mix (Kapa Biosystems). We used an initial denaturation step of 95 °C for 2 min, followed by 33 cycles of 95 °C for 10 s, and 69 °C for 30 s. Melting curves were 95 °C for 15 s (ramping rate 5.5 °C s), 45 °C for 15 s (ramping rate of 5.5°C s^−1^) and 95 °C for 15 s (ramping rate of 5.5°C s^−1^). In the last step of each melting curve, a continuous fluorimetric register was performed by the system at one acquisition register per each degree Celsius. Melting peaks and genotype calls were obtained by using the Eco Real-Time PCR system (Illumina).

### Statistical analysis and meta-analysis

Comparisons of allele frequencies between cases and controls were performed using Chi-square tests. Logistic regression analysis (additive model) was used to adjust for: 1) sex and *APOE* Ɛ4, and 2) age, sex, and *APOE* Ɛ4. Stratification was also conducted according to the presence or absence of the *APOE* Ɛ4 allele. All statistical analyses were performed using PLINK 1.9 software (http://www.cog-genomics.org/plink2/) [27].

Meta-analysis techniques were used to estimate: *ABCA7* rs4147929:G>A and *CD33* rs3865444:C>A effects across studies. Meta-analysis datasets comprise new data generated, samples overlapping with IGAP were excluded (n = 3,994), data from IGAP [9] and available studies published (Supplementary Table 5). Briefly, we carried out a literature search in Pubmed for studies published before March 2018. The search terms were: *ABCA7* and Alzheimer’s disease; and *CD33* and Alzheimer’s disease, respectively. Only studies meeting the following criteria were included: (1) case/control studies evaluating the effect of rs4147929:G>A or rs3865444:C>A markers in AD’s risk; (2) studies that provided an odds ratio with 95% confidence interval as well as the p-value or provide sufficient data to calculate them. Reviews were excluded. We included 1 article for *ABCA7* [28] (32 articles were excluded due to rs4147929:G>A was not genotyped; 1 presented sample overlapping; 66 were not case/control studies and 3 presented limited access). Thus, the meta-analysis sample size for *ABCA7* rs4147929:G>A comprises 182,208 individuals. In case of *CD33*, we included 12 articles [13, 29–39] (6 studies did not genotype rs3865444:C>A; 5 presented sample overlapping; and 43 were not case/control studies). Thus, the meta-analysis sample size for *CD33* rs3865444:C>A comprises 90,913 individuals. Meta-analysis was conducted using the inverse variant method (fixed-effects model) but in the case of heterogeneity, the DerSimonian and Liard method (random-effects model) was used. Heterogeneity was considered significant when *I*^2^ > 50% and p < 0.05. Meta-analysis results and forest plots were generated using OpenMeta (http://www.cebm.brown.edu/openmeta/).

### Linkage disequilibrium analysis using GWAS datasets

Patterns of LD were studied in data from the present study and four non-imputed GWAS datasets: the Alzheimer’s Disease Neuroimaging Initiative (ADNI) longitudinal study [40]; the Genotype-Phenotype Alzheimer Disease Association (GenADA) study [41]; the National Institute of Aging (NIA) Genetic Consortium for Late Onset Alzheimer’s disease study [42]; and the Murcia study [24] (Supplementary Table 6). The genome assembly for the four non-imputed GWAS datasets was NCBI36/hg18.

LD measures (D’ and r^2^) and tests of the significance of LD were calculated between the *APOE* rs429358:C>T or rs7412:C>T markers and *CD33* rs3865444:C>A using Plink 1.9 software (http://www.cog-genomics.org/plink2/) [27] and the R statistics package. LRLD was accepted where D’ ≥ 0.2 and *P* < 0.05. With the objective of discarding LD results generated by chance, we performed bootstrapping analyses; we calculated LD between *APOE* rs7412:C>T or rs429358:C>T and 10,000 random markers, which presented minor allele frequencies (MAF) ≥ 0.20 and ≤ 0.40 (since *CD33* rs3865444:C>A, MAF = 0.30) and that did not localize to chromosome 19.

To further investigate these results, two additional methods were employed. First, the pattern of disequilibrium across the whole of chromosome 19 was calculated according to the methods of Dawson and colleagues [22]; including only markers with MAFs ≥ 0.2. Briefly, we considered 1.7Mb window (1.6Mb overlap) and calculated average values of D' and r^2^ for all marker pairs, which were separated by at least 50kb and at most 500kb. D’ and r2 were calculated using Plink software 1.9 [27]. Second, association analysis was conducted in Plink 1.9 software [27] according to the presence or absence of the Ɛ2 allele. In this second approach, markers in strong LD with the Ɛ2 allele will exhibit stronger associations. P values were adjusted using the Bonferroni correction method.

Unadjusted, stratified, and meta-analysis models were explored (using the methods described above). In the stratification analysis, subjects were classified into three groups: carriers of Ɛ2 or Ɛ4 alleles, and carriers of the Ɛ3Ɛ3 genotype. Ɛ2Ɛ4 genotype carriers were excluded from this analysis.

### Estimation of inbreeding and population structure

Wright’s population inbreeding coefficient (*F*) was calculated according to heterozygote reduction, with regard to Hardy-Weinberg expectations, according to the formulae: *F* = 1 – Ho/He, where Ho is the frequency of heterozygotes observed in the sample population and He is the frequency of heterozygotes expected under Hardy Weinberg. The final inbreeding calculation responds to the mean of *F* per number of markers included in the analysis. Markers were included in this analysis if they were common markers for all the dataset, had MAF > 0.2 [43], and HWE > 0.001. Individuals with less than 99% of available genotypes were excluded. Inbreeding calculations for F.ACE dataset are very imprecise compared to other datasets available, due to it comprises a small number of SNPs. Thus, inbreeding calculation with a higher number of SNPs was also performed excluding F.ACE dataset.

PCs analysis was conducted to discard LRLD was caused by differential population structure. Plink 1.9 software was used to perform the analysis. PCs analysis was conducted in 19,979 markers, which were common between studies, and which presents low linkage disequilibrium (LD) (r2<0.3). In addition, long range LD regions were excluded to the analysis.

### Availability of the data and material

Fundacio ACE datasets used during the current study are available from the corresponding author on reasonable request. Murcia study data analysed are available upon request to Carmen Antunez, Manuel Serrano-Rios and Agustin Ruiz. The GENADA and NIA datasets that support the findings of this study are available from dbGaP but restrictions apply to the availability of these data, which were used under license for the current study, and so are not publicly available. The ADNI datasets analysed during the current study are available in the ADNI repository, http://adni.loni.usc.edu/.

## SUPPLEMENTARY MATERIALS FIGURE AND TABLES




