# Supplementary material for: Genome-wide significant risk factors on chromosome 19 and the APOE locus
Source: Oncotarget. 2018 May 15;9(37):24590–600. doi: 10.18632/oncotarget.25083 (PMC5973862; doi:10.18632/oncotarget.25083)
Supplement: Supplementary file 1 [file oncotarget-09-24590-s001.pdf]

Genome-wide significant risk factors on chromosome 19 and the *APOE* locus

SUPPLEMENTARY MATERIALS

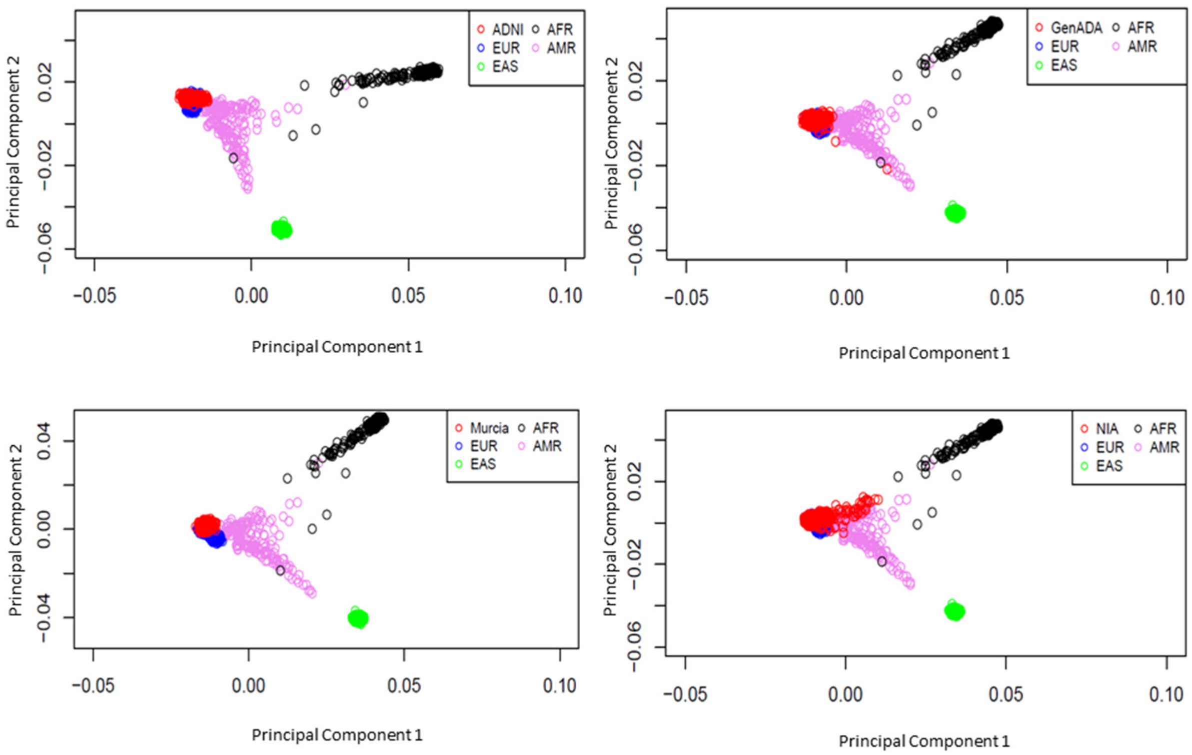

Supplementary Figure 1: Characteristics of the population structure in four analysed GWAS datasets.

Supplementary Table 1: Probability to detect false positive results after LD analysis under specified criterions (D' ≥ 0.2; D' ≥ 0.2 and P ≤ 0.05)

Dataset	P value (D' ≥ 0.2)	P value (D' ≥ 0.2; p < 0.05)
***APOE* rs429358:C>T**		
**ADNI**	0.649	0.058
**GenADA**	0.043	0.002
**Murcia**	0.559	0.011
**NIA**	< 0.001	< 0.001
***APOE* rs7412:C>T**		
**ADNI**	0.383	0.164
**GenADA**	0.132	0.003
**Murcia**	0.150	0.011
**NIA**	0.096	0.002

Supplementary Table 2: Results for the association analysis taking as phenotype the presence of Ɛ2 genotype. *CD33* rs3865444:C>A effect for *APOE*-Ɛ2 phenotype

Dataset	CD33 rs3865444:C>A Effect	Bonferroni Correction (p)	Significant SNPs (Bp range)
**ADNI**	OR =1.44<br>CI =0.79 – 2.62<br>p = 0.23	$9.02x10^{-8}$	2<br>(chr19:50,100,676-50,103,909)
**GenADA**	OR =1.26<br>CI =0.96 – 1.64<br>p = 0.09	$1.14x10^{-7}$	6<br>(chr19:49,868,180-50,103,909)
**Murcia**	OR =0.69<br>CI =0.49 – 0.98<br>p = 0.03	$2.50x10^{-7}$	2<br>(chr19:49,868,180-50,103,909)
**NIA**	OR =0.82<br>CI =0.63 – 1.08<br>p = 0.16	$9.54x10^{-8}$	10<br>(chr19:49,929,652-50,103,909)
**F_ACE**	OR =1.01<br>CI =0.85 – 1.20<br>p = 0.91	NA	NA

Bonferroni Correction (P) = 0.05/SNPs; NA = Not available.
Total number of genome-wide significant SNPs and range of physical distances covered.

Supplementary Table 3: Inbreeding coefficients calculated between common SNPs between datasets and 1000 random SNPs

Dataset	2 SNPS		1000SNPs/1000times		
	N	*F*	N	*F*	SD
**ADNI**	358	-0.019	335	0.002	0.001
**Genada**	1542	0.015	1555	0.004	0.0008
**Murcia**	1052	0.034	972	0.004	0.001
**NIA**	1778	0.017	1778	0.004	0.0007
**F_ACE**	4283	0.009	NA	NA	NA

Supplementary Table 4: Full description of the Spanish replication sample

Dataset	Cases				Controls			
	N	Mean Age (SD)	% Women	%*APOE* ε4	N	Mean Age (SD)	% Women	%*APOE* ε4
**FACE**	1617	82.3 (7.7)	70.4%	45.1%	125	62.3 (7.7)	75.2%	25.6%
**CBC**	159	78.4 (8.5)	69.4%	39.6%	0	-	-	-
**CSC**	0	-	-	-	2002	47.7 (10.6)	50.3%	18.4%
**NXC**	0	-	-	-	515	67.1 (4.7)	96.3%	17.8%
**Murcia**	20	78.4 (8.3)	70%	50%	0	-	-	-
**TOTAL**	1796	82.1 (7.9)	70.2%	44.7%	2642	54.1 (11.6)	64.3%	18.6%

FACE: Fundacio ACE. Institut Català de neurociències (Barcelona, Spain); CBC: Unidad de Memoria, Hospital Universitario La Paz-Cantoblanco (Madrid, Spain); CSC: Proyecto Viva Segovia. Hospital Clínico Sán Carlos; NXC: Neocodex; Murcia: Unidad de Demencias, Hospital Universitario Virgen de la Arrixaca (Murcia, Spain).

Supplementary Table 5: Full description of the datasets used in the meta-analysis assessing *ABCA7* and *CD33* susceptibility in LOAD

See Supplementary File 1

Supplementary Table 6: Number of variants and individuals in non-imputated GWAS datasets

Dataset	N Variants	N cases	N controls	N Total
**ADNI**	553,938	164	194	358
**GenADA**	438,752	782	773	1,555
**Murcia**	198,431	319	769	1,088
**NIA**	524,084	987	802	1,789
