# Supplementary material for: Genome-wide significant risk factors on chromosome 19 and the APOE locus
Source: Oncotarget. 2018 May 15;9(37):24590–600. doi: 10.18632/oncotarget.25083 (PMC5973862; doi:10.18632/oncotarget.25083)
Supplement: Supplementary file 2 [file oncotarget-09-24590-s002.docx]

**Supplementary Table 5.** Full description of the datasets used in the meta-analysis assessing *ABCA7* and *CD33* susceptibility in LOAD.

| ***ABCA7*-rs4147929:G>A Meta-analysis** | | | | | |
| --- | --- | --- | --- | --- | --- |
| **Country/Consortium** | **Study** | **Ancestry** | **N cases** | **N Controls** | **N total** |
| **ADGC** | Lambert 2013 | European | 10,273 | 10,892 | 21,165 |
| **CHARGE** | Lambert 2013 | European | 1,315 | 12,968 | 14,283 |
| **EADI** | Lambert 2013 | European | 2,243 | 6,017 | 8,260 |
| **GERAD** | Lambert 2013 | European | 3,177 | 7,277 | 10,454 |
| **Austria** | Lambert 2013 | European | 210 | 829 | 1,039 |
| **Belgium** | Lambert 2013 | European | 878 | 661 | 1,539 |
| **Findland** | Lambert 2013 | European | 422 | 562 | 984 |
| **Germany** | Lambert 2013 | European | 972 | 2,378 | 3,350 |
| **Greece** | Lambert 2013 | European | 256 | 229 | 485 |
| **Hungary** | Lambert 2013 | European | 125 | 100 | 225 |
| **Italy** | Lambert 2013 | European | 1,729 | 720 | 2,449 |
| **Spain** | Lambert 2013 | European | 2,121 | 1,921 | 4,042 |
| **Sweeden** | Lambert 2013 | European | 797 | 1,506 | 2,303 |
| **UK** | Lambert 2013 | European | 490 | 1,066 | 1,556 |
| **USA** | Lambert 2013 | European | 572 | 1,340 | 1,912 |
| **CCHS** | Kjeldsen 2018 | European | 349 | 9,695 | 10,044 |
| **CGPS** | Kjeldsen 2018 | European | 613 | 93,511 | 94,124 |
| **Fundacio ACE*** | -- | European | 1,500 | 2,494 | 3,994 |
| **Total** | -- | European | 28,042 | 154,166 | 182,208 |
| ***CD33*-rs3865444:C>A Meta-analysis** | | | | | |
| **Dataset** | **Study** | **Ancestry** | **N cases** | **N Controls** | **N Total** |
| **ADGC** | Lambert 2013 | European | 10,273 | 10,892 | 21,165 |
| **CHARGE** | Lambert 2013 | European | 1,315 | 12,968 | 14,283 |
| **EADI** | Lambert 2013 | European | 2,243 | 6,017 | 8,260 |
| **GERAD** | Lambert 2013 | European | 3,177 | 7,277 | 10,454 |
| **Austria** | Lambert 2013 | European | 210 | 829 | 1,039 |
| **Belgium** | Lambert 2013 | European | 878 | 661 | 1,539 |
| **Findland** | Lambert 2013 | European | 422 | 562 | 984 |
| **Germany** | Lambert 2013 | European | 972 | 2,378 | 3,350 |
| **Greece** | Lambert 2013 | European | 256 | 229 | 485 |
| **Hungary** | Lambert 2013 | European | 125 | 100 | 225 |
| **Italy** | Lambert 2013 | European | 1,729 | 720 | 2,449 |
| **Spain** | Lambert 2013 | European | 2,121 | 1,921 | 4,042 |
| **Sweeden** | Lambert 2013 | European | 797 | 1,506 | 2,303 |
| **UK** | Lambert 2013 | European | 490 | 1,066 | 1,556 |
| **USA** | Lambert 2013 | European | 572 | 1,340 | 1,912 |
| **Canada** | Omouni 2014 | European | 524 | 580 | 1,104 |
| **Norway** | Carrasquillo 2011 | European | 346 | 555 | 901 |
| **Poland** | Carrasquillo 2011 | European | 483 | 188 | 671 |
| **USA. CCS.** | Ebbert 2014 | European | 326 | 2,093 | 2,419 |
| **USA.Africans**  **Americans** | Logue 2014 | African American | 513 | 496 | 1,009 |
| **Colombia** | J.D Moreno 2017 | Hispanic American | 280 | 357 | 637 |
| **China. Mainland** | Jiao 2015 | East Asia | 229 | 318 | 547 |
| **China. Ruijin** | Tan 2012 | East Asia | 190 | 193 | 383 |
| **China.Qingdao** | Deng 2012 | East Asia | 612 | 612 | 1,224 |
| **China. WCH** | Zhang 2015 | East Asia | 380 | 475 | 855 |
| **China. Zhejian** | Mao 2015 | East Asia | 126 | 129 | 255 |
| **Korea** | Chung | East Asia | 290 | 554 | 844 |
| **Japan** | Miyashita 2013 | East Asia | 1,008 | 1,016 | 2,024 |
| **Fundacio ACE*** | -- | European | 1,500 | 2,494 | 3,994 |
| **Total** | -- | Heterogeneous | 32,387 | 58,526 | 90,913 |

*Fundacio ACE sample available for meta-analysis excludes samples overlapping with IGAP.
